# Post-COVID-19 Pulmonary Infarction Complicated by Spontaneous Pneumothorax: A Case Report

**DOI:** 10.7759/cureus.26464

**Published:** 2022-06-30

**Authors:** Elisha Taylor, Ivan Novakov

**Affiliations:** 1 Special Surgery, Medical University Plovdiv, Plovdiv, BGR

**Keywords:** post-covid-19 complication, bronchopleural fistula, pulmonary cavitation, pneumothorax, pulmonary infarction, covid-19

## Abstract

Pulmonary cavitation is an atypical finding in COVID-19 patients. In this rare case report, a 63-year-old woman (35 days from COVID-19 symptom onset) presented to our emergency department with acute chest pain and shortness of breath. A chest X-ray established right-sided total pneumothorax, hence a tube thoracostomy was performed. Due to a persistent air leak, chest computed tomography was performed, which showed areas of lung consolidation and a cavitary mass in the upper lobe of the right lung. The woman undertook a thoracoscopy, which established multiple petechiae on the lung surface and a bronchopleural fistula of the right lung’s upper lobe. The treatment of choice was an atypical lung resection to remove the necrotic cavitary lesion. Histological and microbiological examination of the resected lung specimen showed a bland (aseptic) cavitary pulmonary infarct. Pulmonary infarction is a rare cause of cavitation in COVID-19 patients, nonetheless, something that should be considered in those presenting with respiratory symptoms or complications during or post-COVID-19.

## Introduction

Post-COVID-19 is an emerging condition. Definitions are still evolving, and multiple terms are currently being used to describe this period. The National Institute for Health and Care Excellence (NICE) guidelines have defined acute COVID-19 as signs and symptoms up to four weeks from their onset. Signs and symptoms continuing or developing after this time, which are not explained by an alternative diagnosis, are referred to as “Long COVID” [1]. Post-COVID-19 complications are highly variable and can be pulmonary or non-pulmonary. They range from milder, more commonly seen symptoms such as chronic cough and dyspnea-to more severe, rarer complications, including lung infarction and cavitation [1].

Cavitary lung diseases result from various infectious and noninfectious processes [2,3]. A comprehensive meta-analysis noted that lung cavitation is an atypical finding of COVID-19, found in 1.1% of patients [4]. One possible causative factor of lung cavitation in COVID-19 patients is pulmonary infarction [2]. In general, pulmonary infarction is a rare cause of pulmonary cavitation. There are two types of infarct-bland (aseptic) and septic, bland being less common in COVID-19 patients [2].

This paper aims to report a rare case of a post-COVID-19 patient presenting with a bland (aseptic) cavitary pulmonary infarction, complicated by spontaneous pneumothorax.

## Case presentation

A 63-year-old woman presented to our emergency department with an acute onset of mild to moderate chest pain, dry cough, and shortness of breath. The woman had previously had COVID-19 pneumonia, of which symptoms had onset 35 days ago, and was managed and recovered in an outpatient setting. She had neither history of thoracic trauma nor any other comorbidities making her susceptible to pulmonary embolism.

Examination of the chest revealed a decrease of movement on the right side, hyper-resonant percussion, absent tactile fremitus, and diminished breathing sounds on the affected side. A conventional chest X-ray (CXR) established right-sided total pneumothorax with complete lung collapse. The woman was hospitalized, and a right-sided tube thoracostomy was performed. Upon hospitalization, a nasopharyngeal swab was taken and showed a negative PCR result for SARS-CoV-2.

**Figure 1:**
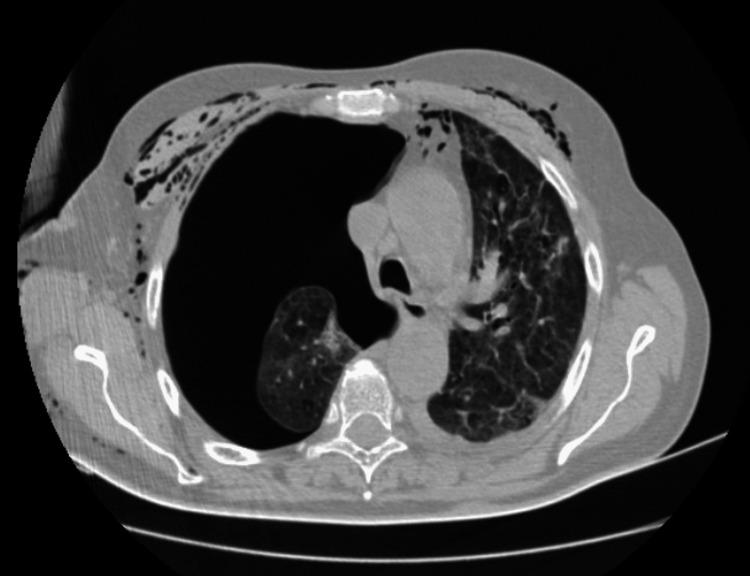
Contrast-enhanced chest CT (4th day of tube thoracostomy) showing no full expansion of the right lung, subcutaneous emphysema, and bilateral areas of small nodular and linear opacities.

**Figure 2:**
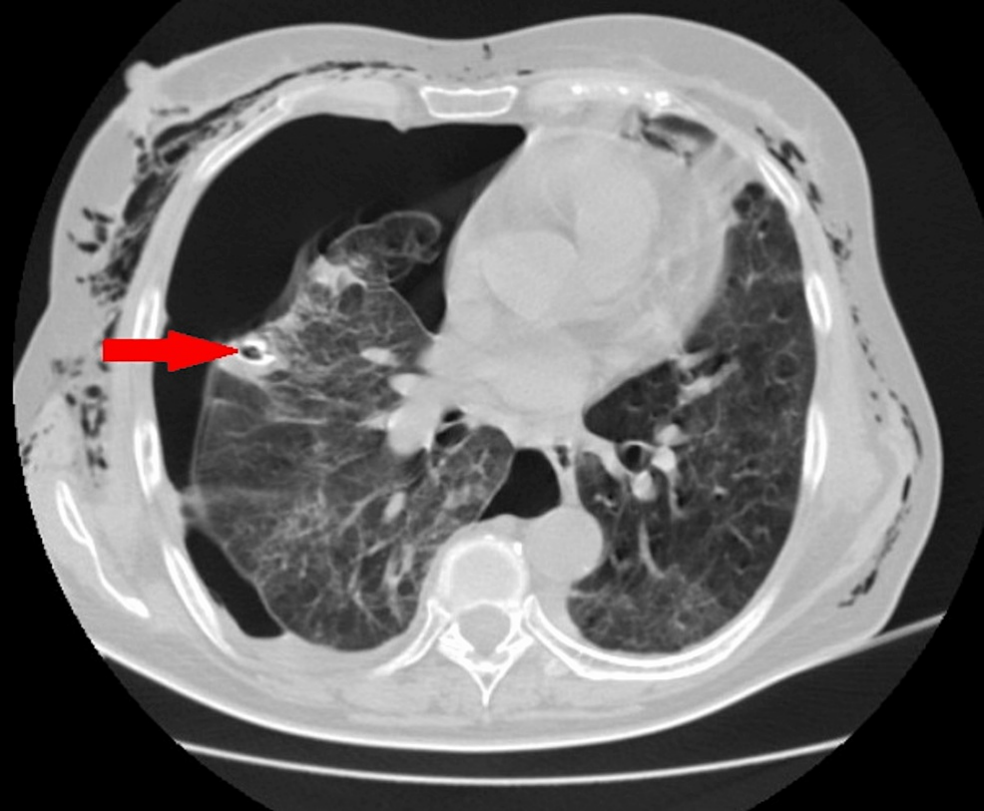
Contrast-enhanced chest CT (4th day of tube thoracostomy) showing a cavitary mass in the right upper lobe (shown by arrow).

Due to a persistent air leak and no full expansion of the right lung, contrast-enhanced chest computed tomography (CT) was performed on the fourth day of tube thoracostomy. The CT showed bilateral areas of lung consolidation with small nodular and linear opacities (Figure 1). A cavitary mass (greatest diameter of 34 mm and maximum wall thickness of 6 mm) in a peripheral, irregular area of consolidation was seen localized in the second segment of the right upper lung lobe (Figure 2). There was no evidence of pulmonary embolism.

Based on chest CT findings, the woman was scheduled for a thoracoscopy that was performed on the sixth day of her hospitalization. Multiple round red spots (petechiae) on the lung surface and a necrotic area (bronchopleural fistula) of the right lung’s upper lobe with leakage of air were established. Due to the thoracoscopic finding, the mini-invasive procedure was converted to a conventional operation (lateral thoracotomy) with atypical resection of the upper lobe of the lung to remove the necrotic cavitary lesion. The intraoperative frozen examination did not reveal malignancy.

**Figure 3:**
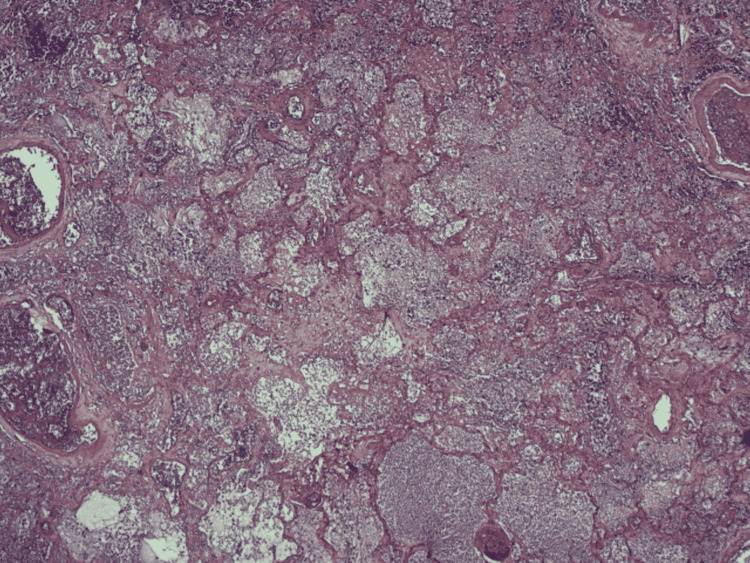
Histological examination of the lung specimen revealed extensive hemorrhagic coagulative necrosis.

**Figure 4:**
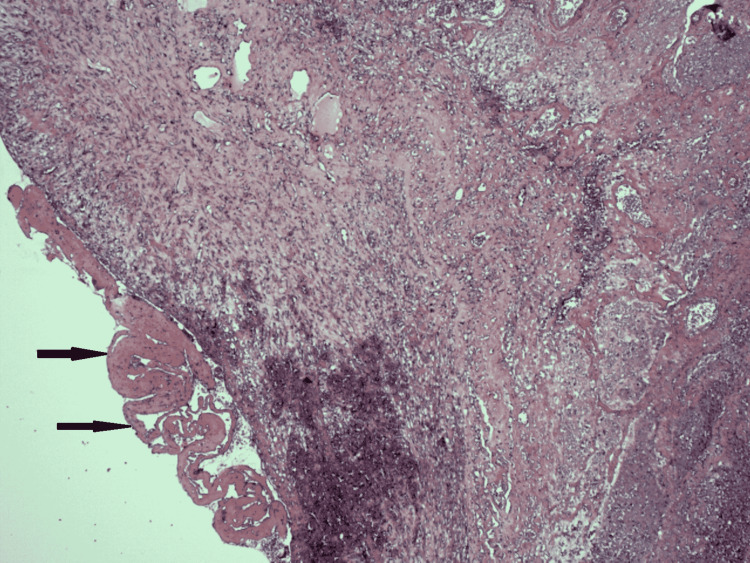
Histological examination of the lung showing margins of the pulmonary infarct lined by a peripheral rim of granulation tissue and deposition of fibrin over the visceral pleura (shown by arrows).

Histological examination of the resected lung specimen showed bland (aseptic) cavitary pulmonary infarction. Subpleural coagulative necrosis of pulmonary parenchyma associated with areas of hemorrhage was seen, with a lining of granulation tissue surrounding the lung necrosis. Outside the granulation tissue were margins of fibrotic tissue with organized and recanalized thrombi and hemosiderin deposition (Figures 3-4). The result of microbiological analysis of the lung specimen was negative, establishing aseptic cavitation.

After thoracotomy, repeated CXRs revealed the right lung was maintaining expansion. The woman was discharged on her ninth postoperative day in good condition.

Written informed consent was obtained from the woman for her anonymized information to be published.

## Discussion

COVID-19 infection is associated with inflammation and endothelial damage, and a hypercoagulable state with an increased risk of thromboembolism, these effects may continue to cause problems post-COVID-19. Pulmonary embolisms, in general, may lead to lung infarction in up to 30% of cases. 3.4-7% of these infarcts may then be complicated with cavitation due to ischemic necrosis of the lung tissue alone (in aseptic pulmonary infarction) or by secondary infection of the necrotic lung tissue (in septic pulmonary infarction). Cavitation may then lead to secondary complications such as empyema, pneumothorax, and bronchopleural fistula [5].

COVID-19-induced pulmonary infarctions with cavitations are rare. However, cavitation in COVID-19 patients, in general, is an under-recognized complication that is now being reported more in hospitals [6,7]. For example, the study from Zoumot et al. shows a 1.7% occurrence of pulmonary cavitation on chest CTs of patients with severe COVID-19 admitted to their hospital, the majority being associated with secondary infections [7]. Unlike in our case-cavitation from an aseptic pulmonary infarction.

Cavitary lung disease can be caused by numerous infectious and noninfectious processes. Among those that should be considered are secondary infections, tuberculosis, fungal infections, necrotizing pneumonia, lung cancer, autoimmune diseases, and, as in our case-ischemic necrosis from pulmonary infarction due to pulmonary emboli and direct endothelial damage of the lung capillaries from COVID-19 infection [2,3]. It is important to note that cavitation in COVID-19 is an atypical finding, not due to the virus itself, therefore should prompt investigations for an underlying cause, alternative diagnosis, and comorbidities [3].

Our patient presented with cough, dyspnea, and chest pain. These are nonspecific symptoms mimicking a typical COVID-19 infection. It is, therefore, important to consider other differentials, such as pulmonary embolism and infarction at admission to prevent misdiagnosis of underlying disease [8]. Contrast-enhanced chest CT is useful in patients with suspicion of cavitation on CXR or COVID-19 complications. In our patient with a pneumothorax, their persistent air leak shown on the mobile suction pump is what led us to order the CT. Upon finding cavitation, further testing, including microbiological analysis should be done to rule out the more common differentials. In our case of pulmonary infarction, histological examination after surgical resection gave us a definitive diagnosis.

One systematic review found the incidence of COVID-19-associated pneumothorax to be 0.3% in hospitalized patients. The majority were associated with more severe COVID-19 infections, particularly in patients requiring mechanical ventilation, causing barotrauma. The study also showed a high mortality rate associated with pneumothorax in COVID-19 patients [9]. Our patient with milder COVID-19, a bronchopleural fistula, and a good postoperative result was distinctive.

## Conclusions

Cavitation in COVID-19 is an atypical finding, therefore alternative diagnoses should be considered. Pulmonary infarction is generally a rare cause of cavitation in COVID-19 patients; this may be further complicated by pneumothorax.

This case was thought to be interesting due to the co-occurrence of numerous uncommon post-COVID-19 complications: aseptic lung infarct, pulmonary cavitation, and bronchopleural fistula with spontaneous pneumothorax and persistent air leak. Clinicians should be aware of COVID-19’s inflammatory effects and prothrombotic state possibly affecting patients beyond acute infection, and causing these post-COVID-19 complications.
